# HIV Treatment Disruption and Viral Suppression Among Venezuelan Immigrants Living with HIV in Miami: A Comparative Analysis

**DOI:** 10.1007/s10461-025-04880-y

**Published:** 2025-09-23

**Authors:** Yue Pan, Viviana E. Horigian, Jorge Saavedra, Elizabeth Alonso, Xinyi Liao, Valeria Botero, Allan E. Rodriguez, Daniel J. Feaster

**Affiliations:** 1 Department of Public Health Sciences, University of Miami Miller School of Medicine, Miami, FL USA; 2 AIDS Healthcare Foundation, Miami, FL USA; 3 Department of Medicine, University of Miami Miller School of Medicine, Miami, FL USA

**Keywords:** Venezuelan immigrants, HIV, Antiretroviral therapy (ART), Viral suppression, Migration, Treatment disruption, Drug resistance, Healthcare access

## Abstract

Venezuelan immigrants living with HIV (VILH) in Miami represent a growing and understudied population that may face substantial barriers to continuous HIV care due to prior treatment disruptions, migration-related stressors, and limited healthcare access. This study compares HIV treatment trajectories, medication histories, and viral suppression among VILH, U.S.-born people living with HIV (PLWH), and other foreign-born PLWH in Miami. We used medical record abstractions from four HIV clinics in Miami. Eligible participants (*n* = 528) were newly enrolled between 2015 and 2019 and classified into three groups: VILH, U.S.-born PLWH, and other foreign-born PLWH. Generalized estimating equations (GEE) were used to assess longitudinal changes in viral suppression and antiretroviral therapy (ART) regimen changes across groups. VILH were more likely to have prior ART exposure (64%) compared to U.S.-born (37%) and other PLWH (33%). Viral suppression improved across all groups, with 89% of VILH achieving suppression at The last visit, compared to 86% among U.S.-born and other PLWH. ART regimen changes and drug resistance testing varied by group, with VILH experiencing more frequent regimen modifications and higher resistance positivity early in care. Significant interactions between migration status and time were observed for both viral suppression and regimen changes (*p* < 0.001). VILH experienced treatment disruptions prior to migration, shown by higher prior ART use, more regimen changes, and increased resistance at care entry. Despite these challenges, VILH achieved comparable viral suppression with sustained care. Ensuring timely and consistent ART regimens for migrants is essential to support treatment continuity and reduce the risk of drug resistance.

## Resumen

Los inmigrantes venezolanos que viven con VIH (IVVIH) en Miami representan una población en crecimiento y poco estudiada que puede enfrentar barreras sustanciales para mantener la atención del VIH de manera continua, debido a interrupciones previas del tratamiento, factores de estrés relacionados con la migración y acceso limitado a los servicios de salud. Este estudio compara trayectorias de tratamiento del VIH, historiales de medicación y supresión viral entre IVVIH, personas nacidas en EE. UU. que viven con VIH (PNVIH) y otros inmigrantes con VIH en Miami. Utilizamos abstracciones de expedientes médicos de cuatro clínicas de VIH en Miami. Los participantes elegibles (*n* = 528) fueron inscritos por primera vez entre 2015 y 2019 y clasificados en tres grupos: IVVIH, PNVIH y otros inmigrantes con VIH. Se emplearon ecuaciones de estimación generalizadas (GEE) para evaluar cambios longitudinales en la supresión viral y en las modificaciones de los regímenes de terapia antirretroviral (TAR) entre grupos. Los IVVIH mostraron mayor probabilidad de haber tenido exposición previa a la TAR (64%) en comparación con los PNVIH (37%) y otros inmigrantes con VIH (33%). La supresión viral mejoró en todos los grupos, con un 89% de los IVVIH logrando supresión en la última visita, frente al 86% de los PNVIH y de otros inmigrantes. Los cambios de régimen de TAR y las pruebas de resistencia a medicamentos variaron entre grupos, observándose en los IVVIH mayor frecuencia de modificaciones de régimen y mayor positividad a resistencia al inicio de la atención. Se identificaron interacciones significativas entre estatus migratorio y tiempo, tanto para la supresión viral como para los cambios de régimen (*p* < 0,001). Los IVVIH experimentaron interrupciones del tratamiento antes de migrar, lo que se reflejó en mayor uso previo de TAR, más cambios de régimen y mayor resistencia al inicio del cuidado. A pesar de estos desafíos, los IVVIH alcanzaron niveles de supresión viral comparables con una atención sostenida. Asegurar el acceso oportuno y consistente a TAR para las personas migrantes es esencial para apoyar la continuidad del tratamiento y reducir el riesgo de resistencia a los medicamentos.

## Introduction

The HIV epidemic continues to disproportionately affect Latinx populations, who represent the largest and one of the fastest-growing ethnic minority groups in the United States and experience higher rates of HIV-related disparities than non-Hispanic White populations [1]. Latinx individuals, particularly recent immigrants, face multiple barriers to HIV care, including lack of health insurance, language barriers, socioeconomic instability, and immigration-related stressors, all of which can contribute to suboptimal antiretroviral therapy (ART) adherence and poor HIV outcomes [2]. Within this broader context, Venezuelan immigrants living with HIV (VILH) have emerged as a particularly vulnerable subgroup. In addition to barriers shared with other immigrants, VILH must also contend with the severe collapse of Venezuela’s healthcare system, including ongoing ART shortages and restricted access to HIV testing [3, 4].

Migration-related stress, including displacement, trauma, legal precarity, and loss of social networks can adversely impact mental health and engagement in chronic care, especially ART adherence [5]. Studies among Latino and other migrant groups have demonstrated that acculturative stress is linked to delays in HIV testing and interruptions in adherence and that multilevel frameworks such as the socioecological model are essential to understand these complex influences on HIV care engagement [6].

Venezuelan migrants living with HIV may face distinct challenges compared to other immigrant groups, driven by the collapse of Venezuela’s healthcare system and uniquely precarious migration circumstances. In a biobehavioral survey of Venezuelan migrants in Colombia, those with irregular migration status had 70% lower odds of viral suppression compared to those with regular status, highlighting the impact of legal precarity on care outcomes [7]. Qualitative research shows that ART scarcity in Venezuela has created structural disruptions, resulting in treatment interruptions and potential drug resistance. These challenges appear more severe than those reported in other Latinx migrant contexts [7, 8]. Together, these vulnerabilities, irregular migration, pre-migration ART shortages, and legal instability, suggest that VILH are likely to experience higher rates of treatment disruption than other foreign-born PLWH newly enrolling in care.

As of 2025, Venezuela’s economy remains in crisis, exacerbated by declining oil revenues and international sanctions. These economic conditions continue to hinder healthcare access, with persistent shortages of ART medications, limited HIV testing, and deteriorating healthcare infrastructure [9, 10]. Public hospitals have struggled to provide consistent HIV care, forcing many PLWH to seek treatment abroad, with Miami emerging as a primary hub for care-seeking Venezuelans [2, 4, 7, 8, 10–12]. Since 2014, an estimated three million Venezuelans have fled The country, constituting about 10% of Venezuela’s population [2]. Among them, a substantial proportion of people living with HIV (PLWH) have sought care in neighboring countries and border cities, with South Florida, particularly Miami, serving as a major point of access to HIV services [2]. According to the Florida Department of Health HIV/AIDS Surveillance Report in 2017, 468 new HIV cases were documented among Venezuelan immigrants in Miami-Dade County [3, 12]. Many of these individuals arrived without continuous access to ART, leading to treatment disruptions and increased risk of viral rebound, HIV drug resistance, and onward transmission [9]. In 2018, Hispanic/Latinx individuals accounted for 70% of new HIV cases in Miami-Dade County, underscoring the public health implications of migration-related HIV care challenges [10].

This study is informed by a socio-ecological framework that considers how structural, community, and individual-level factors interact to shape HIV care outcomes for immigrant populations [13]. The model has been used in previous work to identify multilevel barriers to HIV care in migrant and refugee settings, emphasizing the role of legal, social, and healthcare system contexts in adherence and retention [6, 14, 15].

Migration is a known driver of HIV transmission, as mobility and displacement often lead to interrupted treatment, limited access to healthcare, and increased engagement in high-risk behaviors [10]. Although VILH may benefit from improved healthcare access in Miami, they often experience significant barriers to continuous HIV care, including financial constraints, lack of familiarity with the healthcare system, and delays in securing ART coverage [11]. In Venezuela, healthcare spending has steadily declined, with The government reducing public health expenditures from 9.1% of total spending in 2010 to 5.8% in 2014, leading to severe ART shortages and a collapse of laboratory services [16, 17]. By 2018, most public hospitals in Venezuela had ceased HIV testing, and essential medications such as antiretroviral drugs and condoms were no longer available [18, 19]. Consequently, many PLWH arriving in Miami have experienced prolonged treatment interruptions, leading to higher baseline viral loads, greater susceptibility to opportunistic infections, and increased risk of HIV transmission [20].

Despite the growing number of VILH seeking care in Miami, no study has systematically examined the impact of treatment disruption on HIV virologic suppression and ART regimen changes in this population. This study aims to address this gap by comparing HIV medication history, treatment disruption patterns, and longitudinal viral suppression outcomes among VILH, US-born PLWH, and other foreign-born PLWH newly enrolled in HIV care in Miami. Additionally, this study examines the frequency of HIV medication regimen changes and resistance testing across these groups, as prior non-voluntary treatment interruptions may result in a higher likelihood of ART modifications and resistance surveillance. Given the severe treatment shortages in Venezuela, we expected that VILH would experience more frequent regimen changes and drug resistance testing compared to other groups. Prior ART use among VILH was more uncertain, as systemic shortages limited access in Venezuela; nonetheless, some may have obtained ART intermittently through private markets, humanitarian aid, or treatment abroad. However, with continued engagement in care, all groups are expected to achieve high rates of viral suppression over time, though the trajectory may differ. Findings from this study will provide crucial empirical evidence for developing targeted interventions to improve HIV care continuity for recent immigrants experiencing treatment disruptions.

## Methods

### Study Design and Data Source

This study utilized retrospective medical record abstraction from four major HIV clinics in Miami, including three sites operated by the AIDS Healthcare Foundation (AHF) and one site within the Jackson Health/University of Miami system. Data was collected for individuals who enrolled in HIV care between January 1, 2015, and December 31, 2019. The study design enabled a longitudinal assessment of treatment initiation, medication regimen changes, and viral suppression outcomes, providing insight into the trajectory of HIV care for newly enrolled patients. This study was reviewed and approved by the University of Miami Institutional Review Board (IRB#20200077) with a waiver of informed consent and HIPAA authorization, as the research involved retrospective medical record abstraction without direct patient contact.

### Study Population and Eligibility Criteria

Medical records were abstracted from four major HIV clinics in Miami, Florida, including three AHF-managed clinics and one Jackson Health/University of Miami HIV clinic. The AHF-managed clinics included the South Beach Clinic, Kinder Clinic (located near Mercy Hospital), and Liberty City Clinic. Patients were only included in The study if their first visit to one of these clinics fell within the designated study period of January 1, 2015, to December 31, 2019. The selection of this time frame was intentional, as the Venezuelan economic crisis intensified in early 2015 due to declining oil prices, leading to a large-scale migration of Venezuelans to surrounding countries, including the United States [21].

Eligible participants for this study met the following criteria: They were HIV-positive, as confirmed by medical records at the time of enrollment; their first clinic visit occurred between January 1, 2015, and December 31, 2019; they sought HIV care rather than pre-exposure prophylaxis (PrEP) or other services; and They were at least 18 years old at The time of enrollment. The last available laboratory records were accessed on December 19, 2021. In order to ensure robust comparisons, for each VILH, a non-VILH patient was randomly selected from clinic records to serve as a control group for comparison. To investigate disparities in HIV treatment and virologic suppression, participants were categorized into three groups based on their country of origin. Randomization was conducted using a computer-generated random number assignment applied to the pool of eligible US-born and other foreign-born patients. We did not match participants by demographic factors at the selection stage, because our analytic models adjusted for key demographic and clinical covariates (including age, sex, baseline CD4 count, viral load, and year of enrollment). This approach preserved the representativeness of the underlying clinic populations while minimizing selection bias and addressing potential confounding through multivariable adjustment. The first group included VILH, defined as individuals born in Venezuela who later migrated to Miami. The second group comprised US-born PLWH, referring to HIV patients born in the United States. The third group consisted of other PLWH migrants, representing HIV patients born outside the US, excluding Venezuela. This classification allowed for the examination of differences in HIV care experiences and treatment trajectories across distinct migration backgrounds.

### Measures

#### Primary HIV Care Outcomes

The primary outcomes of interest in this study included HIV viral suppression, HIV medication history, ART regimen changes, and HIV drug resistance testing. These outcomes were selected to assess treatment continuity, potential disruptions, and overall effectiveness of HIV care among different patient groups.

*HIV Viral Suppression* was defined as having a viral load of < 200 copies/mL at three key time points: (1) at the first visit, (2) at the second visit, and (3) at the most recent (last available) visit in the dataset. These time points allowed for the evaluation of early and sustained viral suppression following clinic enrollment.

*HIV Medication History* was assessed based on whether a participant had prior exposure to ART before their first visit (yes/no). This measure captured whether individuals had initiated HIV treatment before enrolling in care at one of the study clinics, which could indicate prior engagement with the healthcare system in their home country or during migration.

*HIV Medication Regimen Change* was assessed to evaluate potential modifications in ART following enrollment. Changes in ART regimens were categorized at three time points: baseline, first visit after baseline, and last visit. A medication change was defined as any modification to the prescribed ART regimen between clinic visits.

*HIV Drug Resistance (HDR)* Testing was evaluated by determining the proportion of patients who underwent resistance testing and the proportion testing positive for drug resistance. This measure provided insight into whether patients had previously experienced ART failure or treatment interruptions that contributed to drug-resistant viral strains.

#### Country of Origin

Participants were classified into three groups based on their country of origin and migration history: US-born PLWH, Venezuelan Immigrants Living with HIV (VILH), and Other PLWH. US-born PLWH were defined as individuals who reported being born in the United States and had Lived there their entire Lives, with no history of migration. Their classification was further validated by ensuring that the majority had received their first HIV diagnosis in the US and had no record of having immigrated from another country. VILH were identified as individuals born in Venezuela who migrated to the US after 2013, corresponding to the period of Venezuela’s economic and political crisis. To qualify as VILH, participants had to both report residence in Venezuela and departure after 2013, and have documentation of an initial HIV diagnosis or prior HIV care in Venezuela before enrolling in The Miami-based HIV clinic. The Other PLWH group comprised individuals born outside the United States and Venezuela who reported a different country of origin. Unlike the VILH group, these participants were not required to have immigrated after 2013. The ‘Other foreign-born PLWH’ group included individuals who immigrated at any time prior to Their enrollment between 2015 and 2019. These classifications were systematically applied to ensure accurate group assignment for comparative analysis.

### Covariates

Demographic Characteristics such as age, gender, and ethnicity (categorized as Hispanic vs. non-Hispanic) were included to assess differences in HIV care engagement and treatment outcomes across patient subgroups.

### Statistical Analysis

Descriptive statistics were used to summarize demographic and clinical characteristics across the three study groups (VILH, US-born PLWH, and other PLWH). Continuous variables, such as age, were presented as means and standard deviations (SD), while categorical variables, such as HIV medication history, medication regimen changes, and drug resistance testing, were reported as counts and percentages stratified by study group and overall. For HIV drug resistance testing, the proportion of participants who underwent drug resistance testing was compared across groups. Among those tested, the proportion who tested positive for resistance was also examined. To evaluate longitudinal changes in viral suppression and medication regimen changes, generalized estimating equations (GEE) models with an exchangeable correlation structure were employed. These models accounted for within-subject correlation across repeated measurements and included interaction terms for country of origin and time, allowing for an evaluation of whether treatment trajectories varied across groups. The GEE models assessed differences in the probability of achieving viral suppression (< 200 copies/mL) at subsequent visits, as well as changes in ART regimens over time. We did not match participants across groups; instead, we adjusted for key demographic and clinical covariates, including age and sex in The GEE models. This regression-based approach is a well-established alternative to matching, typically yielding greater statistical power and retaining the full cohort when sample sizes are moderate to large. To address potential selection bias, sensitivity analyses were conducted to examine the impact of missing viral load data and loss to follow-up on study findings. All statistical analyses were performed using SAS 9.4, with statistical significance set at *p* < 0.05.

## Results

### Demographic and Clinical Characteristics

A total of 528 participants were included in the study (Table 1), comprising 114 (22%) US-born PLWH, 238 (45%) VILH, and 176 (33%) other PLWH. The mean age was similar across groups (US-born PLWH: 37.5 years, VILH: 37.6 years, Other PLWH: 36.9 years). The majority of participants were male (90%), with VILH having the highest proportion (96%) compared to US-born PLWH (81%) and other PLWH (87%). All VILH participants identified as Hispanic/Latino, whereas only 21% of US-born PLWH and 76% of other PLWH did. Enrollment patterns in The HIV care clinics also differed across groups, with the majority of VILH participants enrolling between 2016 and 2018, aligning with the peak migration period following Venezuela’s economic crisis.

**Table 1:** Demographic and clinical characteristics of PLWH enrolled in HIV care clinics in miami, stratified by country of origin (US, venezuela, and other Migrants)

	US (*n* = 114)	Venezuela (*n* = 238)	Other (*n* = 176)	Total (*n* = 528)	*p*-value
Age (Mean, SD)	37.5 (13.3)	37.6 (9.6)	36.9 (11.7)	37.3 (11.2)	0.98
Gender					< 0.0001
Female	22/114 (19%)	10/238 (4%)	23/176 (13%)	55/528 (10%)	
Male	92/114 (81%)	227/238 (96%)	153/176 (87%)	472/528 (90%)	
Ethnicity					< 0.0001
Hispanic or Latino	23/109 (21%)	236/236 (100%)	130/170 (76%)	389/515 (76%)	
Not Hispanic or Latino	86/109 (79%)	0/236 (0%)	40/170 (24%)	126/515 (24%)	
Year enrolled in the clinic					< 0.0001
2014	3/114 (3%)	1/238 (0%)	3/176 (2%)	7/528 (1%)	
2015	13/114 (11%)	20/238 (8%)	24/176 (14%)	57/528 (11%)	
2016	24/114 (21%)	57/238 (24%)	32/176 (18%)	113/528 (21%)	
2017	33/114 (29%)	66/238 (28%)	37/176 (21%)	136/528 (26%)	
2018	28/114 (25%)	62/238 (26%)	42/176 (24%)	132/528 (25%)	
2019	13/114 (11%)	32/238 (13%)	38/176 (22%)	83/528 (16%)	
Pre-baseline HIV/ARV use					< 0.0001
No	60/95 (63%)	83/232 (36%)	113/168 (67%)	256/495 (52%)	
Yes	35/95 (37%)	149/232 (64%)	55/168 (33%)	239/495 (48%)	
Time since last HIV meds before baseline					0.541
Current (0–3 months)	18/30 (60%)	95/144 (66%)	41/53 (77%)	154/227 (68%)	
Past 3–6 months	1/30 (3%)	10/144 (7%)	2/53 (4%)	13/227 (6%)	
Past 6–12 months	2/30 (7%)	7/144 (5%)	3/53 (6%)	12/227 (5%)	
> 12 months	9/30 (30%)	32/144 (22%)	7/53 (13%)	48/227 (21%)	
Viral Suppression at baseline					< 0.0001
Undetectable (< 50 copies/ml)	19/108 (18%)	112/217 (48%)	39/171 (23%)	170/496 (33%)	
Undetectable (< 200 copies/ml)	6/108 (6%)	15/217 (6%)	7/171 (4%)	28/496 (5%)	
Detectable (> = 200 copies/ml)	83/108 (77%)	105/217 (45%)	125/171 (73%)	313/496 (61%)	
Viral suppression at first follow-up					0.3268
Undetectable (< 200 copies/ml)	56/68 (82%)	102/114 (89%)	105/125 (84%)	263/307 (86%)	
Detectable (> = 200 copies/ml)	12/68 (18%)	12/114 (11%)	20/125 (16%)	44/307 (14%)	
Viral Suppression at last follow-up					0.9055
Undetectable (< 50 copies/ml)	64/74 (86%)	183/206 (89%)	125/145 (86%)	372/425 (88%)	
Undetectable (< 200 copies/ml)	5/74 (7%)	11/206 (5%)	8/145 (6%)	24/425 (6%)	
Detectable (> = 200 copies/ml)	5/74 (7%)	12/206 (6%)	12/145 (8%)	29/425 (7%)	

*p-values from one-way ANOVA (age) and chi-square or Fisher’s exact tests (categorical variables), as appropriate

Prior exposure to antiretroviral therapy (ART) varied significantly among the groups (*p* < 0.001). VILH were more likely to have had prior ART use (64%) compared to US-born PLWH (37%) and other PLWH (33%). Among those who had taken HIV medications before Their first clinic visit, treatment interruption patterns differed: 68% resumed ART within 0–3 months, while 21% experienced a gap of more than 12 months. The proportion resuming ART within 0–3 months was highest among other PLWH (77%), followed by VILH (66%), and lowest among US-born PLWH (60%). A treatment interruption of > 12 months was most frequent among US-born PLWH (30%), followed by VILH (22%), and least common among other PLWH (13%). Reasons for ART interruption were not systematically documented in the medical record, limiting our ability to identify specific causes across groups.

### Clinic Enrollment Trends by Year and Country of Origin

**Fig. 1:**
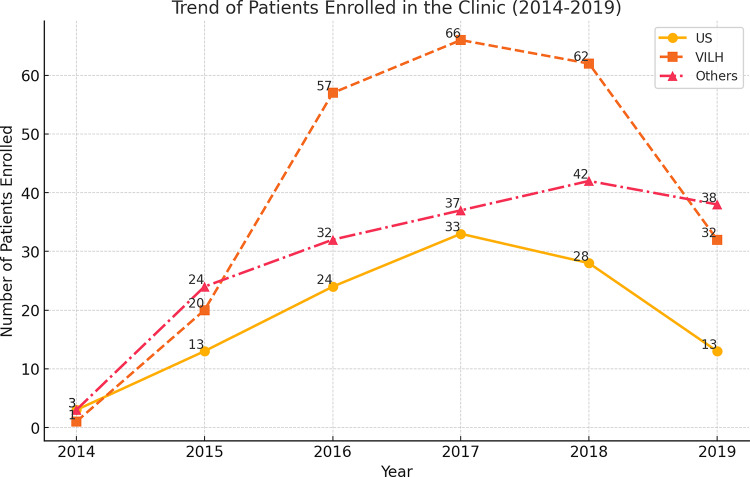
Trend of PLWH enrolled in the Clinic (2014–2019)

The trend of clinic enrollment over time exhibits distinct patterns among US-born PLWH, VILH, and other PLWH (Fig. 1). Enrollment numbers increased steadily from 2014 to 2017, peaking in 2017 and 2018, particularly among VILH, who had The highest number of new enrollees compared to other groups. Enrollment among US-born PLWH and other PLWH followed a similar upward trend but at a more gradual rate compared to the VILH. However, after 2018, a sharp decline in enrollment was observed across all groups, with significantly fewer new patients entering care from 2019 onwards.

### HIV Viral Suppression

At baseline, VILH had the highest proportion with undetectable viral load < 50 copies/mL (48%), significantly higher than US-born (18%) and other PLWH (23%) (*p* < 0.0001). Viral suppression improved over time in all groups, with 86–89% achieving undetectable viral load by the last follow-up. No significant differences in viral suppression at first follow-up (*p* = 0.327) or last follow-up (*p* = 0.906) were observed.

**Fig. 2:**
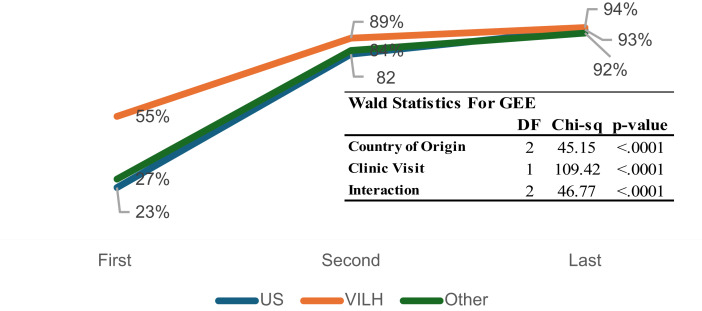
Change in viral suppression (viral load < 200 copies/ml) by country of origin across visits

As shown in Fig. 2, the GEE model confirmed significant improvement over time and by country of origin (Wald χ² = 45.15, df = 2, *p* < 0.0001 for country; χ² = 109.42, df = 1, *p* < 0.0001 for visit). The interaction between country and visit was also statistically significant (χ² = 46.77, df = 2, *p* < 0.0001), indicating that the trajectory of viral suppression varied by migration status. VILH began with a notably higher suppression rate (55%) and reached 94% by the last visit, while US-born and other PLWH started lower (27% and 23%, respectively) but also reached > 90% suppression.

### HIV Medication Regimen Changes

HIV medication regimen changes were assessed over time to evaluate patterns of treatment adjustment (Table 2). At The first visit, 22% of VILH had a medication change, compared to 13% of US-born PLWH and 4% of other PLWH. Over time, The frequency of regimen changes increased across all groups, with 41% of VILH, 37% of US-born PLWH, and 43% of other PLWH experiencing a medication change by their last recorded visit.

**Table 2:** HIV drug resistance of PLWH enrolled in HIV care clinics in miami, stratified by country of origin (US, venezuela, and other Migrants)

	US (*n* = 114)	VILH (*n* = 238)	Others (*n* = 176)	Total (*n* = 528)	*p*-value
Medication Change					
Baseline	12/95 (13%)	40/182 (22%)	7/170 (4%)	59/447 (13%)	< 0.001
First visit after baseline	12/98 (12%)	32/214 (15%)	15/153 (10%)	59/465 (13%)	0.34
Last visit	34/91 (37%)	83/202 (41%)	65/149 (44%)	182/442 (41%)	0.63
HIV Drug Resistance Testing					
Baseline					
Tested	26/114 (23%)	48/238 (20%)	44/176 (25%)	118/528 (22%)	0.5
Positive	5/26 (19%)	4/48 (8%)	5/44 (11%)	9/118 (8%)	0.38
First visit after baseline					
Tested	7/124 (6%)	23/238 (10%)	5/176 (3%)	35/528 (7%)	0.019
Positive	0/7 (0%)	8/23 (35%)	1/5 (20%)	9/35 (26%)	0.17
Last visit					
Tested	2/124 (2%)	2/238 (1%)	4/176 (2%)	8/528 (2%)	0.49
Positive	0/2 (0%)	1/2 (50%)	0/4 (0%)	1/8 (13%)	0.18

*p-values from chi-square or Fisher’s exact tests, depending on cell counts

**Fig. 3:**
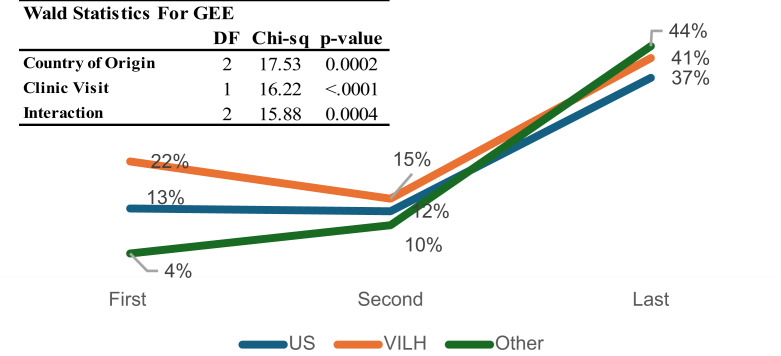
Changes in ART regimen by country of origin across visits

The GEE model revealed significant differences in ART regimen changes across the three groups (χ² = 17.53, df = 2, *p* = 0.0002, Fig. 3). Clinic visit was also significant (χ² = 16.22, df = 1, *p* < 0.0001), as was the interaction term between visit and country of origin (χ² = 15.88, df = 2, *p* = 0.0004). This interaction suggests that the timing and pace of ART regimen adjustments differed by group: VILH started with higher rates of change, dipped slightly at the second visit, and then increased again. In contrast, other PLWH had the lowest initial rate (4%) but experienced The steepest increase, reaching 44% by the last visit. A sensitivity analysis restricted to Latino/Hispanic participants produced results consistent with the main analyses.

### HIV Drug Resistance Testing

Table 2 summarizes HIV drug resistance testing across groups. At baseline, drug resistance testing rates were relatively low (22% overall), with 23% of US-born, 20% of VILH, and 25% of other migrants undergoing testing. Among those tested, drug resistance positivity was detected in 19% of US-born, 8% of VILH, and 11% of other migrants.

At the first visit after baseline, testing rates remained low (7% overall), but resistance positivity was notably higher among VILH participants (35%) compared to US-born (0%) and other migrants (20%). By The last visit, testing rates declined further, with only 2% of participants undergoing drug resistance testing, though one VILH participant (50%) tested positive. Similarly, in a sensitivity analysis restricted to Latino/Hispanic participants, results were consistent with the main findings.

## Discussion

This study provides critical insights into HIV care outcomes among VILH in Miami compared to US-born PLWH and other foreign-born PLWH. Findings reveal significant differences in baseline viral suppression, ART regimen changes, and drug resistance testing across the three groups, highlighting the importance of examining healthcare access within the broader sociopolitical and temporal context, as migration-related treatment disruptions can have lasting effects on HIV outcomes.

### Enrollment Trends and Implications for Healthcare Access

The trend of clinic enrollment over time exhibited distinct patterns among US-born PLWH, VILH, and other PLWH, reflecting broader sociopolitical and healthcare access factors. Enrollment numbers steadily increased from 2014 to 2017, peaking in 2017 and 2018, particularly among VILH, who had the highest number of new enrollees compared to other groups. This peak coincides with the intensification of Venezuela’s political and economic crisis, which led to increased migration and healthcare disruptions, prompting more VILH to seek HIV treatment in Miami [22]. Studies have documented that during this period, Venezuela experienced severe ART shortages, disruptions in HIV services, and a collapse in public healthcare infrastructure, leaving many PLWH with limited treatment options [16, 23, 24].

In contrast, enrollment among US‑born PLWH and other PLWH followed a more gradual upward trend. However, after 2018, a decline in new enrollments was observed across all groups, with fewer new patients entering care from 2019 onwards. This decline may be attributed to policy changes affecting migration and healthcare access, including the expansion of the “public charge” rule in 2019, which broadened the criteria for inadmissibility and created a chilling effect on immigrants’ use of Medicaid and other services; and intensified immigration enforcement policies such as family separation under the “zero‑tolerance” policy in 2018, which generated fear and mistrust within immigrant communities [25, 26]. These shifts likely deterred healthcare engagement and enrollment in HIV care, compounding natural declines following earlier waves of Venezuelan migration. These trends underscore the significant influence of sociopolitical policies on healthcare access among migrant populations and highlight the need for targeted interventions to ensure sustained HIV care during periods of policy volatility.

### HIV Viral Suppression and Treatment Trajectories

At baseline, VILH exhibited a higher proportion of viral suppression compared to US-born PLWH and other PLWH. This may be attributed to a greater likelihood of prior ART exposure among VILH, as indicated by their higher rates of prior ART use before clinic enrollment. However, despite this advantage, VILH also had more frequent ART regimen changes and higher drug resistance positivity at the first visit after baseline, suggesting that many had experienced unstructured treatment interruptions before migration, leading to potential virologic failure or ART resistance. These findings align with prior research showing that prolonged ART interruptions can result in viral rebound and an increased risk of developing drug-resistant HIV strains [19, 20]. Given the well-documented ART shortages in Venezuela [16, 23, 24] many VILH may have relied on irregular medication access or inconsistent dosing schedules before arriving in Miami, contributing to their higher likelihood of ART regimen changes and drug resistance.

The longitudinal analyses demonstrated significant improvements in viral suppression across all groups over time, with VILH achieving rates comparable to US-born and other PLWH by the last visit. The significant interaction effect between country of origin and clinic visit suggests that treatment trajectories differed by migration status, with VILH experiencing a steeper improvement curve. This likely reflects the benefits of consistent access to ART in Miami, reinforcing the importance of sustained HIV care engagement for newly arrived immigrants. This result is particularly relevant given the increasing migration trends from Venezuela due to political and economic instability. Previous studies have shown that migration-related barriers, including financial insecurity and lack of familiarity with the healthcare system, can delay ART initiation and result in worse health outcomes for PLWH [18, 23, 24]. Despite these challenges, our study suggests that once engaged in care, VILH can achieve similar levels of viral suppression as other groups, emphasizing the importance of accessible and immigrant-friendly HIV care services.

### HIV Medication Regimen Changes

Frequent HIV medication regimen changes can indicate challenges in achieving optimal treatment due to previous interruptions, drug resistance, or adverse effects. While ART regimen adjustments are sometimes necessary to optimize treatment outcomes, frequent modifications may also reflect underlying treatment instability. Findings from our study highlight the role of migration-related healthcare disruptions in influencing ART regimen changes, particularly among VILH, who may have experienced non-voluntary treatment interruptions before enrollment in care.

The need for ART modifications in this population could be driven by several factors, including prior inconsistent access to medication in Venezuela, the potential for drug resistance due to interrupted treatment, and differences in available ART regimens between countries. Additionally, recent immigrants may face challenges in navigating the U.S. healthcare system, resulting in delays or complications in medication management. The initial differences in ART changes between groups may reflect these disparities in treatment continuity. However, over time, as patients established consistent care, the frequency of ART modifications converged across groups, suggesting stabilization of treatment.

Ensuring ART regimen stability is essential for long-term HIV outcomes. Studies have shown that frequent regimen changes are associated with lower adherence and an increased risk of virologic failure, which could compromise treatment success and public health efforts to reduce HIV transmission [20, 27, 28]. These findings underscore the importance of targeted interventions to support treatment continuity among newly arrived immigrants with a history of disrupted care.

### HIV Drug Resistance Testing

HIV drug resistance testing is a critical component of effective treatment management, particularly for individuals with a history of treatment interruptions. The findings from this study indicate that while drug resistance testing rates were relatively low overall, VILH had notable differences in resistance positivity compared to US-born and other PLWH. The higher resistance rates observed in VILH may be attributable to prolonged treatment gaps before migration, as ART shortages in Venezuela have been well documented [12, 22]. Interruptions in ART can lead to the development of resistance mutations, particularly in individuals previously treated with first-line regimens that have lower genetic barriers to resistance [20, 27, 28].

Despite these initial differences, resistance testing rates declined over time across all groups. This trend may reflect improved treatment adherence and viral suppression with continued engagement in HIV care, reducing the clinical necessity for repeated drug resistance testing. However, the low overall testing rates suggest potential gaps in routine surveillance, which may result in undetected resistance mutations and suboptimal ART regimens for some patients.

Given the high risk of treatment interruptions among recent migrants, routine drug resistance testing at baseline and during early stages of care could be beneficial in guiding ART selection. This approach may help clinicians identify individuals at risk of virologic failure due to pre-existing resistance mutations and ensure that they are placed on the most effective regimens from the outset. Future research should explore strategies to integrate systematic resistance testing for newly enrolled PLWH with prior ART exposure, particularly in migrant populations from regions with known ART shortages. Ensuring early and accurate resistance detection is essential for preventing further transmission of drug-resistant HIV strains and improving long-term virologic outcomes.

### Implications for Practice and Research

These findings have important implications for clinical practice. The elevated baseline suppression rates among VILH likely reflect a history of ART exposure, yet their higher rates of regimen changes and resistance suggest unstructured treatment interruptions before migration. Clinicians should be aware that recent immigrants may present with complex ART histories and may require additional adherence support, regimen adjustments, and early resistance testing to optimize treatment outcomes.

From a public health and research standpoint, our results reinforce the value of continuity of care in mitigating the effects of prior treatment disruptions. Despite disparities at baseline, VILH achieved comparable suppression rates by the end of the observation period, highlighting that high-quality, sustained HIV care can overcome migration-related setbacks. Future research should evaluate structural interventions, such as health system navigation, language-concordant services, and legal aid to support the care engagement of newly arrived immigrants.

Additionally, the significant interaction effects observed in both viral suppression and ART regimen changes suggest that migration history is not only associated with baseline disparities but also modifies longitudinal care trajectories. These findings emphasize the need to disaggregate foreign-born populations when evaluating HIV outcomes, as patterns may vary widely depending on pre-migration healthcare access, sociolegal context, and resettlement conditions.

Finally, integration of HIV care with immigration, housing, and social services may be critical in supporting recent arrivals who are highly vulnerable to gaps in care. Policies ensuring early linkage to care and routine monitoring, including baseline resistance testing, could enhance treatment outcomes and reduce disparities. At the policy level, our findings underscore the importance of immigrant-sensitive healthcare policies in both Miami and Venezuela. In Miami, local and state policies that expand Medicaid eligibility, strengthen Ryan White program support, and reduce barriers tied to immigration status could mitigate treatment disruptions for newly arrived immigrants. In Venezuela, the ongoing collapse of the public health system highlights the urgent need for international partnerships and aid programs to stabilize ART supply chains and laboratory services. Coordinated policy responses on both sides, strengthening the safety net in receiving countries like the U.S., while supporting healthcare system recovery in Venezuela, are essential to sustaining viral suppression and preventing the spread of resistant HIV strains. Additionally, future work should investigate how differences in immigration status (e.g., asylum seekers, undocumented individuals) influence ART adherence and access, particularly in light of shifting federal and state policies.

### Strengths and Limitations

This study has several strengths. First, it utilizes real-world clinical data from four major HIV care sites in Miami, providing a comprehensive assessment of treatment outcomes in a diverse patient population. Second, the study applies a rigorous classification criteria to distinguish VILH from other foreign-born and US-born PLWH, ensuring that findings accurately reflect migration-related disparities in care. Third, the longitudinal study design allows for an evaluation of changes in treatment trajectories over time, offering valuable insights into the impact of continuous care engagement on viral suppression and ART regimen stability.

However, there are also limitations. First, the study relies on retrospective medical record abstraction, which may be subject to incomplete or missing data. While sensitivity analyses were conducted to assess potential selection bias, unmeasured confounders could still influence findings. Second, drug resistance testing was not performed systematically across all patients, leading to potential underestimation of resistance prevalence. Third, while the study includes a large sample of VILH, findings may not be generalizable to all Venezuelan immigrants living with HIV, particularly those who remain outside of healthcare systems. Third, the sample was predominantly male (approximately 90%), which limits generalizability to female or gender-diverse populations. Sex-related vulnerabilities, including differences in treatment adherence, ART side effects, and sociostructural barriers to care may affect the extent to which these findings apply to women or other groups. Fourth, while the study includes a large sample of VILH, findings may not be generalizable to all Venezuelan immigrants living with HIV, particularly those who remain outside of healthcare systems. Fifth, the data reflect the 2015–2019 period, and although this may not capture the most recent policy shifts or migration trends, it corresponds to the peak of the Venezuelan migration crisis and provides a unique window into treatment disruptions during a time of severe ART shortages. Many of the structural barriers faced by VILH during that period, including healthcare access challenges, legal precarity, and immigration-related stressors, remain highly relevant, underscoring the continued applicability of our findings. Lastly, structural barriers such as immigration status, employment, and access to health insurance were not fully accounted for in the analysis, despite their known impact on HIV care engagement [18, 20, 29–31].

## Conclusion

This study highlights the challenges faced by VILH in maintaining continuous HIV treatment before migration, as evidenced by their higher rates of prior ART use, frequent regimen changes, and elevated ART resistance at initial care entry. However, with sustained engagement in Miami’s HIV care system, VILH achieved comparable viral suppression rates to other groups, reinforcing the importance of ensuring uninterrupted access to ART for newly arrived immigrants. As the migration of PLWH from crisis-affected regions continues globally, clinicians, public health officials, and policymakers must prioritize immigrant-sensitive strategies to close care gaps and prevent the emergence of drug resistance. Given the continued migration of PLWH to the United States, future research should explore the long-term impact of treatment disruptions on health outcomes among VILH and identify targeted interventions to support their integration into the healthcare system.
